# Papillary fibroelastoma of the aortic valve presenting with chronic angina and acute stroke: a case report

**DOI:** 10.1186/s13256-016-1179-x

**Published:** 2017-01-18

**Authors:** Fan Zhang, Ziqiang Zhu, Gautham K. Upadhya, Jiankun Tong, Vlad Gotlieb, Abdullah Khan, Rakesh P. Gupta

**Affiliations:** 1 Department of Internal Medicine, Brookdale University Hospital and Medical Center, One Brookdale Plaza, Brooklyn, NY 11212 USA; 2 Department of Pathology, New York–Presbyterian/Queens, Flushing, NY 11355 USA; 3 Division of Hematology/Oncology, Brookdale University Hospital and Medical Center, One Brookdale Plaza, Brooklyn, NY 11212 USA; 4 Division of Cardiology, Brookdale University Hospital and Medical Center, One Brookdale Plaza, Brooklyn, NY 11212 USA; 5 Division of Cardiology, New York–Presbyterian/Queens, Flushing, NY 11355 USA

**Keywords:** Cardiac tumor, Papillary fibroelastoma, Angina, Acute stroke, Aortic valve, Case report

## Abstract

**Background:**

Papillary fibroelastomas are rare, benign cardiac tumors that are often found on cardiac valvular surfaces. Most are incidental discoveries during surgery or autopsy. The clinical presentation of fibroelastoma varies widely, ranging from clinically asymptomatic to severe thromboembolic events.

**Case presentation:**

We report a case of 65-year-old white man diagnosed with scattered, bilateral acute cerebral hemisphere infarcts with a history of chronic angina. Transesophageal echocardiography identified a fibroelastoma on the right coronary cusp of the aortic leaflet. Cardiac catheterization revealed mild non-obstructive stenosis. We postulate that the etiology of his angina is related to the dynamic occlusion of his right coronary ostium by the fibroelastoma.

**Conclusions:**

To the best of our knowledge, this is the first case report describing a patient with a cardiac papillary fibroelastoma who presented with both chronic angina and acute stroke.

## Background

Papillary fibroelastoma (PFE) is the second most common benign primary tumor of the heart [1]. The clinical presentation of PFEs varies widely, ranging from primarily asymptomatic to severe ischemia with embolic events. PFEs usually involve the cardiac valves and are now being recognized more frequently with the aid of transesophageal echocardiography (TEE). Symptomatic cardiac PFEs and asymptomatic, mobile left-sided lesions greater than 1 cm in diameter should be evaluated for surgical excision [2]. There is an excellent postoperative prognosis with no recurrences having been reported to date. We describe a rare case of a 65-year-old man, with a history of chronic angina, who presented with an acute stroke and was incidentally found to have a PFE on the right coronary cusp of the aortic leaflet.

## Case presentation

A 65-year-old white man with a history of hyperlipidemia, hypertension, and chronic angina, presented with a sudden onset left-sided visual field deficit with left upper extremity weakness. The symptoms started abruptly while he was working at his computer. On arrival at an Emergency Department (ED), his visual symptoms had resolved, but he still had residual weakness and difficulty coordinating his left upper extremity. A physical examination revealed a body mass index (BMI) of 40.5 kg/m^2^, blood pressure of 147/82 mmHg, partial left-sided hemianopia, 4/5 strength in left arm/hand with pronator drift, and normal heart sounds. A chest X-ray revealed a mildly enlarged cardiac silhouette. Computed tomography (CT) of his head without contrast was unremarkable. His National Institutes of Health Stroke Scale (NIHSS) was 3 and he was within the 3-hour window, thus tissue plasminogen activator (tPA) was given without delay. However, his symptoms did not improve. He denied any similar prior episodes or any family history of premature coronary artery disease and/or stroke. He had a previous cardiac stress test which was negative.

**Fig. 1:**
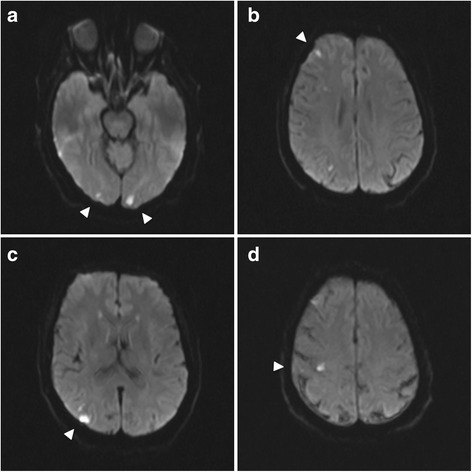
Magnetic resonance imaging of the brain. Scattered bilateral cerebral hemispheric small cortical-based acute infarcts (arrows) with a distribution suggestive of embolic phenomenon and alternatively watershed regions, including right occipital (a, c), left occipital (a), right frontal (b), and right parietal lobes (d)

**Fig. 2:**
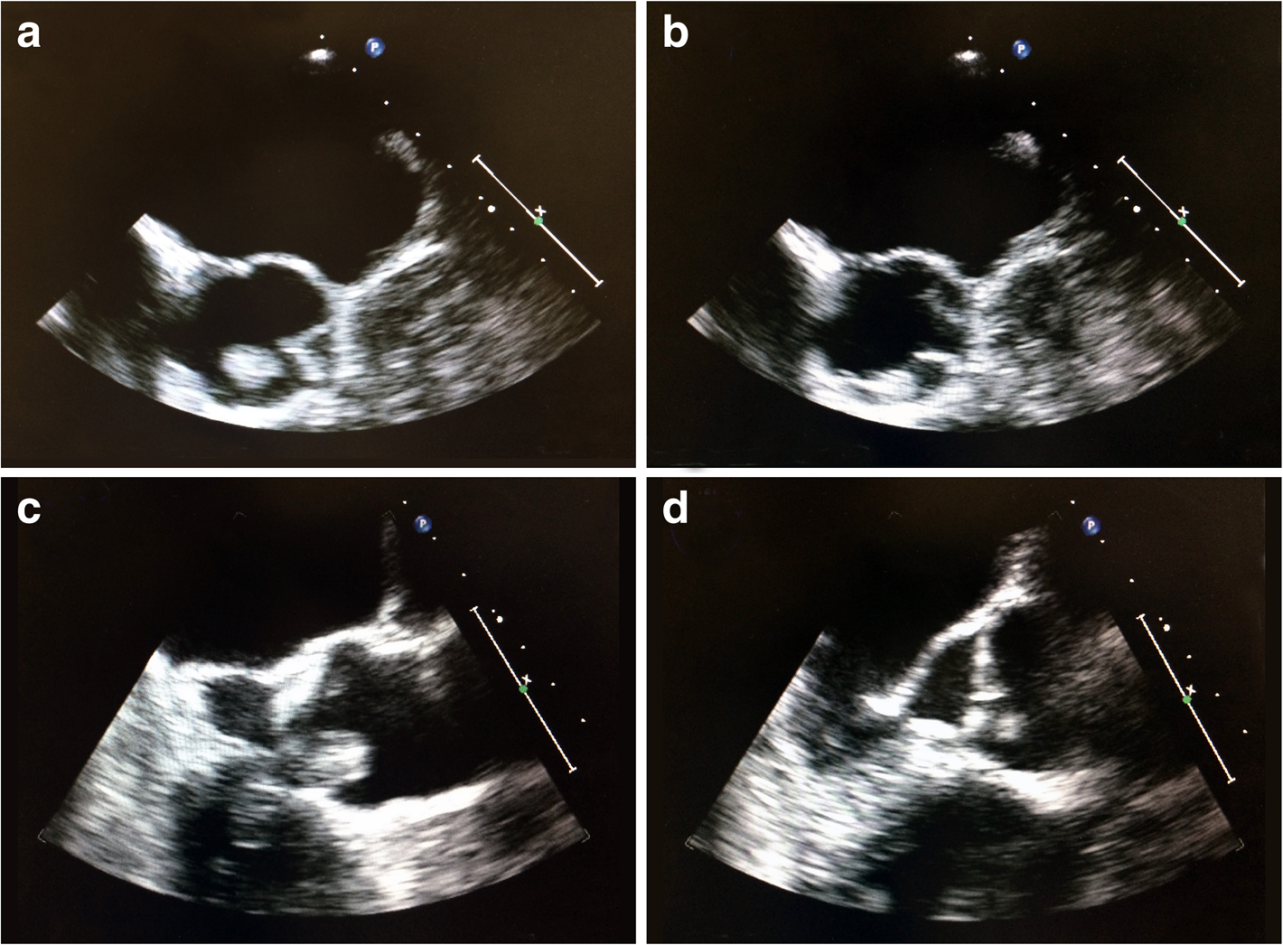
Transesophageal echocardiography. **a**, **b** Transesophageal echocardiography in the mid-esophageal view of aortic valve at long axis revealed a mobile mass 11×15 mm in diameter attached to the right leaflet of the aortic valve. It partially prolapsed in the left ventricular outflow tract of the aorta. **c**, **d** Mid-esophageal aortic valve short axis view of the papillary fibroelastoma attached to the aortic side of the right coronary cusp

**Fig. 3:**
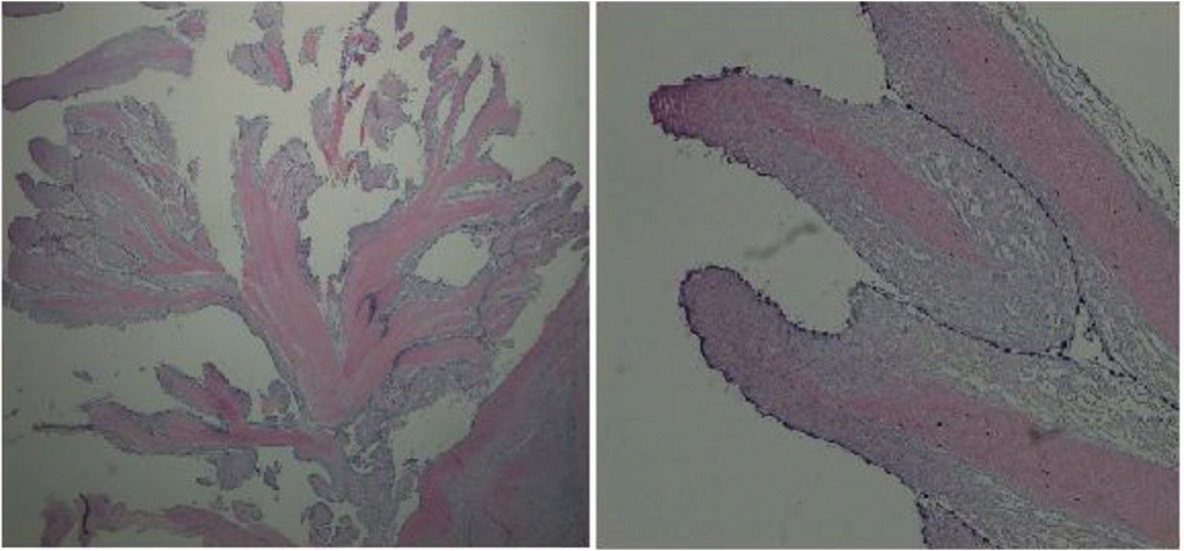
Hematoxylin and eosin stain of the papillary fibroelastoma with narrow elongated and branching papillary fronds formed by central avascular collagen and elastic tissue core and the flat endocardial lining (*left panel* view, 2× magnification; *right panel*, 10× magnification)

Further workup with magnetic resonance imaging (MRI) of his brain discovered small, scattered, bilateral, cortically based acute infarcts with a distribution pattern suggestive of an embolic event. The scattered areas had T2 hyperintensities in the right frontal, parietal, and occipital regions, all with associated diffusion and apparent diffusion coefficient (ADC) map abnormalities (Fig. 1). Transthoracic echocardiography (TTE) noted a small rounded echodensity on the right coronary cusp of the aortic leaflet. Repeat TEE reported structurally normal aortic valves with a round, pedunculated mobile mass measuring approximately 11×15 mm, attached to the right coronary leaflet (Fig. 2). The appearance of the mass was characteristic of a PFE. Prior to surgical removal of his PFE, our patient underwent left-sided cardiac catheterization owing to his history of angina and multiple surgical risk factors. The results of which revealed normal coronary arteries except for mild luminal irregularities with proximal and mid-segment stenosis (20%) of his left anterior descending (LAD) artery. He eventually underwent a bioprosthetic aortic valve replacement with excision of the leaflets and mass. The mass was soft, pink-yellow in color, and measured 1.3×1.0×0.7 cm. It had narrow, elongated papillary fronds and a hyalinized central core surrounded by flat endocardial lining. Pathology results confirmed PFE with myxoid degenerative changes (Fig. 3). He developed transient postoperative atrial fibrillation which was initially controlled by amiodarone before reverting to a normal sinus rhythm. He was discharged to short-term rehabilitation and has made a successful recovery.

## Discussion

PFEs are rare cardiac tumors with a prevalence of 0.002 to 0.28% among the general population [3]. The average age at diagnosis is 56, with a primarily male preponderance (58%) [4]. As a primarily benign endocardial tumor, most are reported because of advances in clinical imaging. PFEs are the most common valvular tumors of the heart, accounting for 10% of all cardiac primary neoplasms [1]. Clinical presentation varies depending on the location, dimensions, growth rate, mobility, and tendency for embolization. The masses are generally asymptomatic and are discovered incidentally during surgery and/or autopsy. Even though PFEs are histologically benign, they can result in serious complications. The most common clinical manifestation of a symptomatic fibroelastoma is a transient ischemic attack and/or stroke [5]. Other manifestations include cardiac angina, myocardial infarction, sudden cardiac death, heart failure, presyncope or syncope, pulmonary embolism, blindness, mesenteric ischemia, peripheral emboli, and renal infarction.

Cardiac PFEs arise most often from the left side of the heart, frequently in clinical association with hypertension. They have a high propensity to affect the aortic valve (44%), the left ventricular outflow tract and the anterior mitral leaflet (35%). However, PFEs have been observed on all valves (84%), and even with occasional formation within the mural endocardium [1]. Single or multiple lesions can develop. Among patients with aortic valve tumors, sudden death and myocardial infarction were the two most common outcomes [6]. The mitral valve was reported to be the most common source of systemic tumor embolization, such as in stroke [7]. Rarely, when present on the aortic valve, PFEs may cause angina by transiently occluding the coronary ostia and/or by embolizing into the coronary arteries. In our case, the patient presented with both stroke and angina.

PFEs affect coronary artery blood flow and can present as exertional chest pain, suggestive of chronic angina [7], or acute coronary syndrome [8]. Of interest, in cases where PFEs affected coronary artery blood flow, the PFEs were found to be attached to the right coronary cusp. The aortic valve has three cusps: left, right, and non-coronary. The ostium of the right coronary artery is found above the right coronary cusp. The ostium of the left main coronary artery is found midway between the commissures of the left coronary cusp, while the non-coronary cusp is positioned posteriorly. PFEs usually present as mobile ball-like tumors located at the top of the right coronary cusp. They often contain pedicles with multiple papillary fronds which vary in dimensions ranging from 2 to 50 mm in length. In our case, the patient had a history of chronic angina with a prior work up including a negative nuclear stress test as well as a cardiac catheterization revealing insignificant stenosis. Therefore, the chronic angina was probably due to transient obstruction of the coronary ostia by PFEs located on the right coronary cusp.

Structurally, PFEs resemble chordae tendineae. They are avascular tumors composed of an outer endothelial layer, and a dense central core composed of a rim of loose mucopolysaccharide-rich connective tissue, dendritic cells, fibroblasts, and smooth muscle cells [1]. On gross examination, PFEs appear to have multiple frond-like projections that look like sea anemone attached to the endocardium [3]. The matrix consists of proteoglycans and prominent elastic fibers that form a concentric pattern, contiguous with the underlying valve leaflet, which was confirmed with immunohistochemical staining [9].

The high embolic potential of PFEs is due to the friability of their tissue matrix in addition to their extreme mobility within the aortic root. The intermittent dislodgement of papillary frond fragments and/or the platelet/fibrin thrombi formed on them may lead to thromboembolic events, such as in acute coronary syndrome and cerebrovascular accidents (CVAs) [10].

The exact mechanisms leading to the development of PFEs are still unclear. There are several hypotheses involving organizing thrombi, congenital hamartomas, iatrogenic formations, cytomegalovirus infection, rheumatic valve disease, as well as true primary neoplasms [11]. The most widely accepted explanation is that of the microthrombus theory. This presumes that the tumor is formed by minor endothelial damage on the margins of the valves, which serves as a nidus for the growth and progression of microthrombi. These then coalesce into overgrowths similar to those of Lambl’s excrescences [12]. However, unlike Lambl’s excrescences, which localize at valvular closing lines and the free edges of valve cusps, PFEs are found on any type of endocardial tissue. PFEs can grow to diameters of up to 1 to 5 cm, compared to Lambl’s excrescences which are much smaller [13]. This theory is also supported by the location of PFEs on non-valvular endocardial surfaces close to prior cardiac procedure sites or radiation fields up to 18 years later [14].

It is important to differentiate PFEs from cardiac myxomas and thrombi because of the differences in their medical treatment. A clear surgical margin is necessary for the excision of a myxoma due to its high rate of recurrence. On the other hand, fibroelastomas rarely recur after resection, thus it is recommended to preserve valvular function by shaving off the tumor [15]. Although TTE is widely used to screen for PFEs, TEE is more sensitive and can provide higher resolution imaging for surgical planning and intraoperative guidance, which lead to changes in management in 16.7% of patients with suspected cardioembolic stroke [13].

On echocardiography, PFEs typically appear as homogenous speckled, round, mobile, pedunculated or sessile masses, mostly located on cardiac valves [16]. In contrast, myxomas are heterogenous masses with broad-based pedicles and little mobility, predominately in the left atrium [17]. Lambl’s excrescences are more numerous, smaller, and broader-based lesions located near the lines of valvular closure [1]. Although thrombi can be differentiated by their irregular shape, laminated appearance and absence pedicles [18], native aortic valve thrombi often resemble PFEs, which can lead to unnecessary surgical interventions [16]. Bacterial vegetations are more irregular in appearance [19]. The development of cardiovascular magnetic resonance (CMR) and multidetector-row CT (MDCT) as tools in the evaluation of soft tissue masses of the heart is currently under investigation.

PFEs carry a very high risk of thromboembolic complications including CVA and cardiac events, such as in this case. The only independent predictor of PFEs-related deaths and/or nonfatal embolizations is tumor mobility. Asymptomatic immobile tumors with diameters less than 1 cm can be followed closely by clinical evaluation and echocardiography. While urgent surgical resection is recommended for all symptomatic patients who have mobile pedunculated tumors that are increasing in size. Surgical resection via a valve-sparing shave is curative and safe. However, in the case of advanced cardiac valve involvement, such as in our case, valve replacement should be considered. Among affected patients, it has been reported that 83% were treated with simple tumor resection, 9% with tumor resection and valve repair, and 10% required prosthetic valve replacement. Symptomatic patients who are not surgical candidates may be observed closely and offered therapeutic anticoagulation.

## Conclusions

We report a rare case of a PFE located on the right coronary cusp that is associated with both a CVA as well as chronic angina. These presentations are due to the mobility and friability of the mass, which cause transient occlusion of the right coronary orifice in addition to intermittent dislodgement of papillary fragments. The differential diagnosis includes cardiac myxoma, Lambl’s excrescences, bacterial vegetations, and thrombi. Echocardiography with TEE is more sensitive for guidance. PFEs are resectable and carry an excellent postoperative prognosis as well as a low recurrence rate. Long-term cardiologic surveillance is recommended.

## Abbreviations

ADC: Apparent diffusion coefficient
BMI: Body mass index
CMR: Cardiovascular magnetic resonance
CT: Computed tomography
CVA: Cerebrovascular accident
ED: Emergency Department
LAD: Left anterior descending
MDCT: Multidetector-row computed tomography
MRA: Magnetic resonance angiogram
MRI: Magnetic resonance imaging
NIHSS: National Institutes of Health Stroke Scale
PFE: Papillary fibroelastoma
TEE: Transesophageal echocardiography
tPA: Tissue plasminogen activator
TTE: Transthoracic echocardiography
